# Compliance with dietary guidelines affects capillary recruitment in healthy middle-aged men and women

**DOI:** 10.1007/s00394-015-1151-3

**Published:** 2016-01-08

**Authors:** Virginia Govoni, Thomas A. B. Sanders, Dianne P. Reidlinger, Julia Darzi, Sarah E. E. Berry, Louise M. Goff, Paul T. Seed, Philip J. Chowienczyk, Wendy L. Hall

**Affiliations:** 1 Diabetes and Nutritional Sciences Division, Faculty of Life Sciences and Medicine, King’s College London, 4.108 Franklin-Wilkins Building, 150 Stamford Street, London, SE1 9NH UK; 2 Women’s Health Division, King’s College London, St Thomas’ Hospital, London, UK; 3 British Heart Foundation Centre, School of Medicine, King’s College London, St Thomas’ Hospital, London, UK

**Keywords:** Dietary guidelines, Cardiovascular disease, Microcirculation, Capillaroscopy, Blood pressure, Randomized controlled trial

## Abstract

**Purpose:**

Healthy microcirculation is important to maintain the health of tissues and organs, most notably the heart, kidney and retina. Single components of the diet such as salt, lipids and polyphenols may influence microcirculation, but the effects of dietary patterns that are consistent with current dietary guidelines are uncertain. It was hypothesized that compliance to UK dietary guidelines would have a favourable effect on skin capillary density/recruitment compared with a traditional British diet (control diet).

**Methods:**

A 12-week randomized controlled trial in men and women aged 40–70 years was used to test whether skin microcirculation, measured by skin video-capillaroscopy on the dorsum of the finger, influenced functional capillary density (number of capillaries perfused under basal conditions), structural capillary density (number of anatomical capillaries perfused during finger cuff inflation) and capillary recruitment (percentage difference between structural and functional capillary density).

**Results:**

Microvascular measures were available for 137 subjects out of the 165 participants randomized to treatment. There was evidence of compliance to the dietary intervention, and participants randomized to follow dietary guidelines showed significant falls in resting supine systolic, diastolic and mean arterial pressure of 3.5, 2.6 and 2.9 mmHg compared to the control diet. There was no evidence of differences in capillary density, but capillary recruitment was 3.5 % (95 % CI 0.2, 6.9) greater (*P* = 0.04) on dietary guidelines compared with control.

**Conclusions:**

Adherence to dietary guidelines may help maintain a healthy microcirculation in middle-aged men and women. This study is registered at www.isrctn.com as ISRCTN92382106.

## Introduction

Functional and structural modifications in the microcirculation occur early in the development of hypertension and diabetes [1–9], and it has been suggested that a reduction in the number of perfused microvessels at rest eventually leads to a loss of anatomical microvessels altogether (structural rarefaction) as well as functional impairment of capillary perfusion and recruitment [1, 2]. Measuring microcirculation in the skin is recognized as a predictive, non-invasive marker of generalized microvascular and endothelial function [10], with studies showing impaired microvascular function to be concomitantly present in skin, coronary [11] and kidney [12] vessels. Skin microcirculation can be measured as capillary density, defined as the number of perfused vessels for unit area of tissue (mm^2^), and can be distinguished as functional or structural. Functional density reflects the number of vessels perfused at rest, while structural density represents the total number of vessels present but only perfused when the metabolic demand of the tissue is greater [13], which is simulated by inflating a finger cuff to stop venous flow and measuring the density again after a few minutes. The difference between the two is called capillary recruitment (CR) and can be expressed as a percentage increase.

The restoration of microvascular function could help decrease systemic blood pressure (BP) by reducing peripheral vascular resistance, which in turn could improve circulation in target organs and help decrease cardiovascular disease (CVD) complications [14]. Evidence for the effect of diet on microcirculation is limited. The majority of published studies report the impact of single dietary components, such as salt [14, 15], omega-3 fatty acids [16] and polyphenols [17–19], in improving microvascular function. Current UK dietary guidelines (DG) for the prevention of CVD focus on maintaining a healthy weight, a reduction in the intakes of saturated fat, salt and added sugar, and increased consumption of fruit, vegetables, wholegrains and oily fish [20]. The aim of this study was to determine the effect of following a diet consistent with DG on skin capillary density/recruitment in men and women aged 40–70 years, compared with a traditional British dietary pattern.

## Participants and methods

The CRESSIDA study (Cardiovascular disease risk REduction Study), registered at www.isrctn.com as 92382106, was a single-centre dietary intervention trial with a randomized, parallel design to test the effects of 12-week adherence to DG [21], compared with a traditional British dietary pattern (CON), on BP, vascular function and total cholesterol/HDL cholesterol ratio. The primary outcomes have been reported and showed significant reductions in daytime ambulatory systolic BP [−4.2 mmHg (95 % CI −6.6, −1.7; *P* < 0.001)] and TC/HDL-C molar ratio [−0.13 (−0.26, 0.00; *P* = 0.044)] in the DG group compared to the CON group [22]. The study was conducted at King’s College London between August 2010 and July 2012, approved by South London NHS Research Ethics Committee (Ref: 10/H0802/24), and received NHS R&D approval from the Guy’s and St Thomas’ Hospitals Foundation Trust.

Detailed methodology for laboratory analysis, diet formulation and study design have been previously published [22].

### Participants

Volunteers were recruited through newspaper advertisements and electronic internal circulars to staff and students at King’s College London. All subjects gave written informed consent at the first screening visit, whereupon BP and anthropometric measurements were made, and urine and blood samples were collected for assessment of non-smoking status (urinary cotinine), and fasting blood lipid profile, glucose, liver function and haematology were assessed to confirm eligibility to take part in the study. The main inclusion criteria were: healthy and non-smoking men and women between the age of 40 and 70 years, while the exclusion criteria included a reported history of CVD, type 1 or type 2 diabetes mellitus, use of medication for lowering blood cholesterol or BP, body mass index (BMI) <18.5 and >35 kg/m^2^, an overall risk of CVD over the next 10 years >20 % estimated using the QRISK software (QRISK.org), clinical history of cancer (excluding basal cell carcinoma) in the past 5 years, chronic renal, liver or inflammatory bowel disease, history of substance abuse or alcoholism, current self-reported weekly alcohol intake exceeding 21 U for women and 28 U for men, pregnancy or having had a baby in the last 12 months. Furthermore, subjects were excluded if they reported being unwilling to follow the protocol and/or give informed consent, to refrain from use of dietary supplements, to restrict consumption of oily fish in accordance with the allocated diet or if they had a weight change of >3 kg in the preceding 2 months.

### Study design

Before randomization to treatment, eligible participants were asked to complete 2 × 24-h ambulatory BP measurements (results reported previously [22]) and urine collections, a 4-day food record, and validated food-frequency questionnaires. Treatment was allocated by minimization for age, sex, ethnicity and BMI by using a custom-designed computer database (MedSciNet AB). Participants attended the clinical research facility at St Thomas’ Hospital, London, UK, in the fasting state for measurements of vascular function, including video-capillaroscopy, and to provide blood samples; procedures were repeated after 12 weeks. Full dietary advice based on the randomly allocated diet was given at baseline by a registered dietitian, and dietary assessments and lifestyle information were collected by questionnaires and 4-day food diary at baseline and at study endpoint.

### Dietary intervention

The intervention diet (DG) was based on UK Department of Health dietary guidelines [21]. Targets for the DG group included reductions in the intake of total fat and saturated fatty acids to <35 and <11 % food energy, respectively, a reduction in added sugars to <11 % food energy, a reduction in salt intake to <6 g/day, and an increase in fruit and vegetable, wholegrains and oily fish intake to ≥5, ≥2 portions/day and ≥1 portions/week, respectively. The control diet (CON) was nutritionally balanced and based on a traditional British diet, formulated with familiar foods (full-fat milk, cheese, butter, meat and meat products, non-wholegrain cereals), and which reflected typical intakes of fruit and vegetables (3 portions/day), oily fish (<1 portion/month), saturated fatty acids (~13 % energy) and did not restrict intakes of salt or added sugar. Participants were counselled by a dietitian and provided with tailored advice. Both DG and CON groups were provided with some foods to aid compliance including pasta, microwavable rice, breakfast cereals, tinned beans and fish, nuts, cooking oils and spreads. Food intake records were checked and measurements of biomarkers of compliance were made to verify that the participants were following the advice, including measurements of 24-h urinary sodium, potassium, and sucrose plus fructose excretion as markers of salt, fruit and vegetables, and added sugars intake, respectively, and percentage changes in long chain n-3 PUFA in red blood cell membrane lipids (biomarker of oily fish intake) and plasma alkylresorcinols (biomarker of wholegrains intake).

### Measurement of skin capillary density and capillary recruitment

Microvascular measures using skin video-capillaroscopy were secondary outcomes of the CRESSIDA study [22]. Measures were taken in accordance with standard operating procedures (SOPs) developed within the British Heart Foundation Centre at St Thomas’ Hospital, based on published methodology and following best practice in vascular measures [6, 8, 13, 23]. Capillary densities were measured on the dorsum of the middle phalanx of the third finger of the left hand, or fourth finger if rings were present and not removable, using the CAM1 Capillary Anemometer and CapiScope™ System (KK Research Technology Ltd., Devon, UK) in a temperature-controlled room (mean 22 ± 1.8 °C). Analyses were carried out in the morning in a fasted state; prior to this, participants consumed standardized low-fat/low-salt meals the evening before. After 30-min supine rest, measurements were made of supine BP in triplicate at 5-min intervals using the Omron 705CP device. Following this, measurements of carotid–femoral pulse wave velocity, radial pulse wave analysis and digital volume pulse wave were made, and after a further 20 min, capillary measurements were made. The left arm was supported at heart level in a comfortable position with the finger positioned under the microscope and kept in place with a plastic finger clamp to limit movement. The site of skin to be analysed was covered with drops of vegetable oil to limit light reflection. Measurements were taken at baseline (before dietary intervention at week 0) and following the 12-week dietary intervention.

Four fields were identified around a central point and recorded for 10 s each before and after venous occlusion. This was achieved by inflating a small finger cuff positioned at the base of the finger to 60 mmHg for 5 min. Total numbers of capillaries in each video, visible as black dots on a grey background, were counted offline using the CapiScope™ automatic software and manually corrected for movement artefacts. Functional capillary density (FCD) was calculated from the average density of the four fields taken at rest, and structural capillary density (SCD) as the average density taken after 5 min of cuff inflation. Densities are reported as number of vessels per mm^2^.

Capillary recruitment was derived by subtracting FCD values from the SCD and expressing it as a percentage. Reproducibility was tested in ten healthy subjects before starting the intervention. The day-to-day reproducibility of the technique was 4.2 % for FCD and 3.2 % for structural CD with correlation coefficients of 0.9 (*P* = 0.001) for both measures. Within-day intra-observer reproducibility was also acceptable, with a CV of 7.1 % for FCD and 5.9 % for SCD.

### Statistical analysis

Analyses were performed with Stata version 13.1 (Stata Corp). All variables were checked for normality. Variables that were not normally distributed were natural log-transformed, and the data expressed as geometric mean. Data were analysed on a per protocol basis by analysis of covariance with the endpoint value regressed against the baseline value, gender, age and BMI categories; the treatment effect is the regression-adjusted difference between groups. Causal mediated effect analysis, which estimates the extent to which the specified effect of the randomized treatment is mediated through its other effects, was conducted to explore whether the changes in CR could be explained by changes in sodium excretion, BMI and systolic BP [24].

## Results

**Fig. 1:**
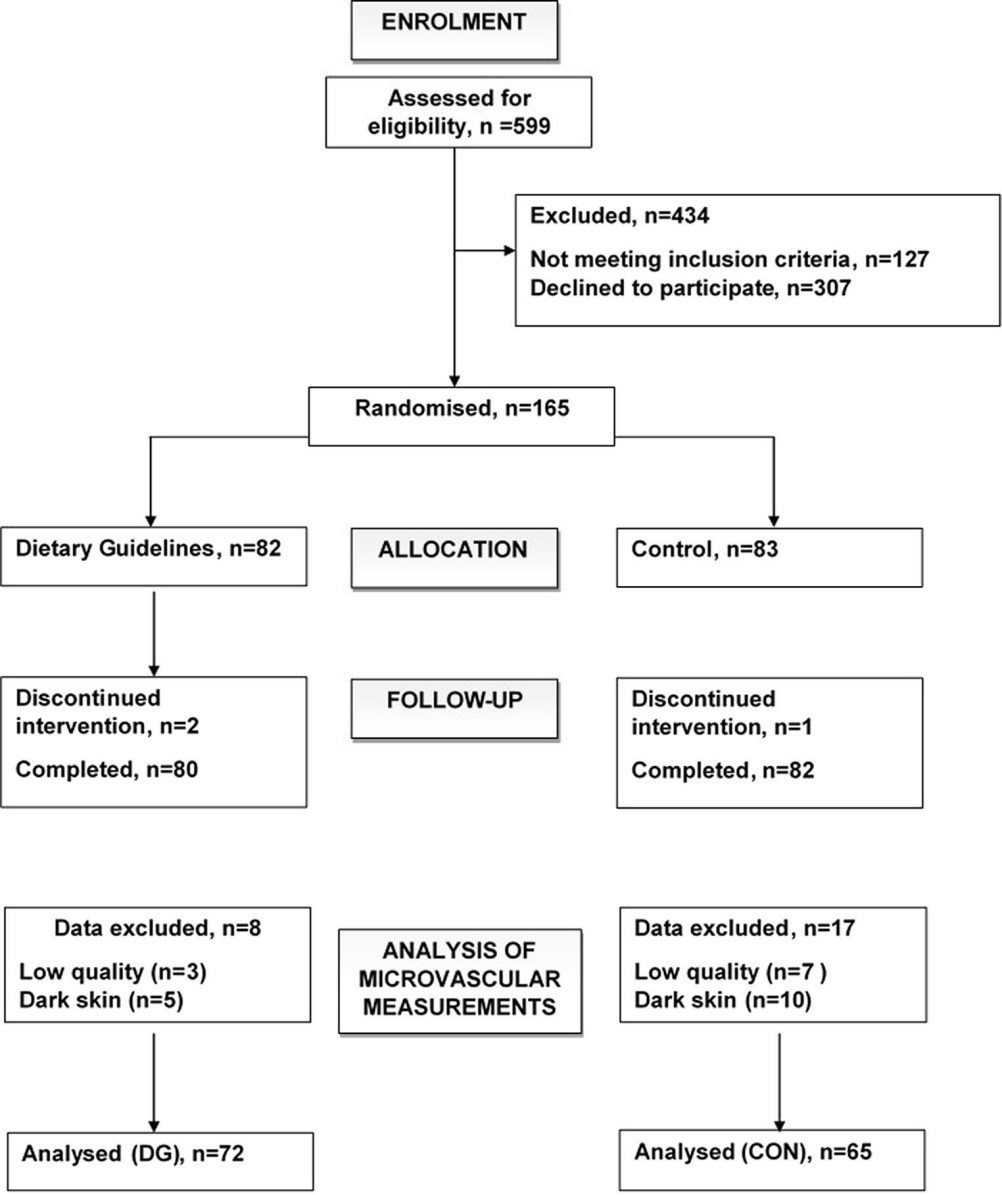
Consort diagram

Out of 165 participants randomized to treatment, 162 participants completed the study and data were available for analysis in 137 participants (72 DG, 65 CON). Data were missing for 15 participants because the technique could not be performed on darkly pigmented skin and on ten participants due to missing measurements. Figure 1 shows the flow of participants through the study, and Table 1 shows their characteristics. The participants were on average aged 53 years old with a BMI of 26 kg/m^2^. They were predominantly normotensive (seated BP < 140/90 mmHg) and of white European origin. Non-smoking status was confirmed by urinary cotinine analysis. Over half of the women were post-menopausal. Table 2 reports the main dietary goals and the biomarkers used to check compliance to the diets. There was no evidence of differences between groups in their baseline dietary intakes or baseline characteristics. Compliance to dietary advice on both DG and CON diets was verified by reported dietary intakes and analysis of biomarkers. Saturated fatty acid intakes were reduced to target levels in 95 % of the DG group, and this was accompanied by a 10 % fall in low-density lipoprotein (LDL) cholesterol as would be expected following reduced saturated fatty acid intakes. Decreases in urinary sucrose and fructose excretion in the DG group indicated compliance to advice to reduce intake of added sugars. An increase in wholegrain consumption on DG was evidenced by increased plasma alkylresorcinol concentrations. Compliance to oily fish advice was demonstrated by increases in the proportions of eicosapentaenoic (20:5n-3) and docosahexaenoic (22:6n-3) acid in erythrocyte lipids. Reported fruit and vegetable intakes were already close to target at baseline, an observation that was supported by urinary potassium excretion which was generally high. Although reported fruit and vegetable servings per day were increased in the DG group compared to CON, there was no significant difference between groups in terms of potassium excretion, an indication that all participants were consuming plenty of fruit and vegetables. In addition to lower dietary sodium intakes estimated from 4-day food diaries, urinary sodium excretion was markedly reduced by the DG intervention.

**Table 1:** Details of male and female participants by randomized treatment group

	DG	CON
	*n* = 72	*n* = 65
Age	53 ± 8	53 ± 8
*Sex*		
Male, *n* (%)	31 (43)	26 (40)
Pre-menopausal female, *n* (%)	15 (21)	15 (23)
Post-menopausal female, *n* (%)	26 (36)	24 (37)
*Ethnicity*		
White European, *n* (%)	66 (92)	61 (94)
Asian, *n* (%)	6 (8)	4 (6)
BMI (kg/m^2^)	25.0 ± 3.3	26.5 ± 3.7
Fasting plasma total cholesterol (mmol/L)	5.3 ± 1.0	5.3 ± 0.9
Fasting plasma HDL cholesterol (mmol/L)	1.6 ± 0.4	1.5 ± 0.5
Fasting plasma TG (mmol/L)	1.0 ± 0.6	1.2 ± 0.5
Fasting plasma glucose (mmol/L)	5.3 ± 0.5	5.2 ± 0.4
Seated systolic BP (mmHg)	118 ± 14	119 ± 13
Seated diastolic BP (mmHg)	76 ± 8	78 ± 9
10-year risk of CVD men (%)	7.6 ± 5.3	7.9 ± 5.7
10-year risk of CVD women (%)	3.7 ± 3.1	4.9 ± 3.4

Mean ± SD. 10-year risk of CVD estimated using QRISK-2. No significant differences between groups: two-sample *t* test or Mann–Whitney *U* test

*DG* dietary guidelines, *CON* control, *BMI* body mass index, *BP* blood pressure, *CVD* cardiovascular disease, *HDL* high-density lipoprotein

**Table 2:** Comparison of dietary intakes estimated from 4-day food diaries at baseline and following intervention, together with biomarkers of compliance on treatment, compared with targets for the dietary guidelines group

	Target^$^	Baseline	DG	CON
		*n* = 137	*n* = 72	*n* = 65
Total fat (% food energy)	≤35	35.7 ± 5.9	34.0 ± 5.3*	37.7 ± 5.3
SFA (% food energy)	<11	12.1 ± 3.3	7.7 ± 1.8*	15.0 ± 2.8
LDL-cholesterol (mmol/L)		3.2 ± 0.85	3.0 ± 0.77*	3.3 ± 0.78
NMES (% food energy)	<11	9.7 ± 4.4	7.1 ± 4.0*	9.4 ± 3.8
24-h urinary sucrose and fructose (μmol/24 h)		41 (33, 50)	28 (21, 38)*	56 (43, 73)
Wholegrains (servings/day)	≥2	1.4 ± 1.1	2.7 ± 1.36*	1.0 ± 1
Plasma alkylresorcinol (nmol/L)		75 (66, 85)	87 (74, 102)*	64 (53, 78)
Oily fish (servings/week)	≥1	1.0 ± 1.0	1.7 ± 1.56*	0.2 ± 0.3
Erythrocyte 20:5n-3 + 22:6n-3 (wt%)		7.1 ± 2.2	8.0 ± 2.4*	6.7 ± 2.1
Fruit and vegetables (servings/day)	≥5	5 ± 3	7 ± 3*	5 ± 3
24-h urinary potassium (mmol/day)		83 ± 29	86 ± 30	81 ± 22
Sodium intake (mmol/day)	<100	142 ± 53	84 ± 83*	152 ± 59
24-h urinary Na excretion (mmol/day)		125 ± 55	80 ± 39*	133 ± 55

Mean values ± SD or geometric mean (95 % CI)

There were no statistical differences between DG and CON at baseline

*DG* dietary guidelines, *NMES* non-milk extrinsic sugars

* Significant difference from control *P* < 0.01; two-sample *t* test

^$^140 g oily fish = 1 serving; 30 g wholegrains = 1 serving; 80 g fruit or vegetable = 1 serving

### Effect on blood pressure and microcirculation

Supine peripheral systolic, diastolic and mean arterial BP all fell significantly (all *P* < 0.001) on the DG compared with CON by 3.4, 2.5 and 2.9 mmHg, respectively (Table 3). There were no significant differences in functional or structural capillary density at baseline, but following the intervention, CR was 3.5 % greater following DG compared with CON which was statistically significant (*P* = 0.04). This significant difference remained after adjusting for changes in mean arterial pressure and baseline sodium excretion. Causal mediated effect analyses did not find significant effects for changes in urinary sodium excretion or changes in weight.

**Table 3:** Changes in supine blood pressure (BP) and digital microcirculation in healthy non-smoking men and women aged 40–70 years before and 12 weeks after random allocation to dietary guidelines (DG) or control (CON) diets

	DG (*n* = 72)	CON (*n* = 65)	Treatment effect	*P* value
*Supine systolic BP* (mmHg)				
Baseline	117 (114, 120)	116 (113, 119)		
Endpoint	112 (110, 115)	115 (113, 118)	−3.5 (−5.4, −1.7)	<0.001
*Supine diastolic BP* (mmHg)				
Baseline	74 (72, 76)	74 (72, 76)		
Endpoint	71 (69, 73)	74 (72, 76)	−2.6 (−3.8, −1.4)	<0.001
*Supine mean arterial BP* (mmHg)				
Baseline	88 (86, 90)	88 (86,90)		
Endpoint	85 (83, 87)	88 (86, 90)	−2.9 (−4.2, −1.6)	<0.001
*Functional capillary density* (n/mm^2^)				
Baseline	110 (106, 113)	110 (105, 114)		
Endpoint	108 (104, 112)	102 (102, 110)	1.1 (−2.8, 5.1)	0.57
*Structural capillary density* (n/mm^2^)				
Baseline	116 (111, 120)	113 (109, 118)		
Endpoint	118 (113, 122)	113 (109, 116)	2.5 (−0.8, 5.9)	0.14
*Capillary recruitment* (% increase)				
Baseline	6.4 (4.3, 8.4)	3.7 (1.9, 5.6)		
Endpoint	9.7 (7.3, 12.1)	6.5 (4.3, 8.6)	3.5 (0.2, 6.9)	0.04

Mean values (95 % CI). Data were analysed on a per protocol basis, and the probabilities are based on analysis of covariance with value on treatment regressed against the baseline value, gender, age and BMI categories; the treatment effect is the regression-adjusted difference between groups

## Discussion

Adherence to DG increased post-occlusive CR in the skin relative to CON, suggesting improved regulation of blood flow in the microcirculation. The differences between treatment groups were marginal, and definitive conclusions may only be drawn once further studies have confirmed these effects. Improved capillary function may have been an indirect consequence of reduced peripheral resistance which increases tissue perfusion. BP-lowering drugs (ACE inhibitors, angiotensin-II receptor antagonist and some diuretics) improve microvascular function in both animals [25] and humans [26], with mechanisms including activation of bradykinin pathways, stimulation of vascular endothelial growth factor production and increased nitric oxide formation. However, as previously reported, we were unable to demonstrate any effect of diet on nitric oxide-dependent vasorelaxation of the brachial artery [22], nor measures of radial pulse wave augmentation index or digital volume pulse reflection index (unpublished data). The improvement in skin CR following the DG diet compared to a traditional British diet appeared to be independent of the changes in BP.

He et al. [14] reported skin capillary microcirculation data from a crossover trial of slow sodium (sodium chloride tablets in slow release form) compared to placebo, with each intervention lasting 6 weeks in 169 hypertensive subjects following a reduced salt diet (mean BP 147/91 mmHg). Salt intake on slow sodium was 9.7 g/day (159 mmol/day) compared to 6.5 g/day (111 mmol/day) on placebo. FCD was significantly lower following slow sodium treatment compared to placebo (101 and 106 capillaries per mm^2^, respectively), and the same was observed for SCD following 2-min venous congestion (108 and 115 capillaries per mm^2^, respectively). In the present study, the duration of intervention was longer, but CRESSIDA participants were non-smokers and mainly normotensive. These factors may explain the discrepancy with the findings of He and colleagues as regards the lack of effect observed on resting capillary density with an integrated dietary intervention that included salt restriction. The capillary densities in our participants were similar to the values reported by He et al. in hypertensives following sodium restriction (106 capillaries per mm^2^ FCD and 115 capillaries per mm^2^ SCD) and are similar to values reported in other studies in predominantly normotensive individuals [8, 27–30]. Furthermore, it should be noted that the microvascular measures were a secondary outcome of the current study, and the possibility that the study was underpowered to detect changes in capillary density cannot be excluded.

A significant novelty of the study is that the dietary intervention comprised a whole diet approach. Evidence in healthy populations showing that adherence to combined DG might modify markers of risk of CVD is scarce. Most studies focus on single components of dietary advice and are often conducted in high-risk populations or populations with CVD. The present study was conducted in non-smoking individuals at average risk of CVD who were not receiving medication for lipids and BP. The study included a relatively large sample size and a relevant duration for observing changes in vascular function. Furthermore, good compliance to the dietary intervention was achieved as evidenced by validated biomarkers of dietary compliance and the falls in BP. Other than a slightly higher intake of saturated fat (by 8 g/day), the CON diet was nutritionally balanced and was not markedly different from the participants’ usual diet. The study indicates the expected size of impact on microvascular function to be expected in a healthy middle-aged population adhering to DG. It was not possible to attribute through causal mediated effect analysis the components of the diet that were responsible. This may have been due to a lack of statistical power. Alternatively, the effect may be the sum of several components of the intervention.

An important limitation of the capillaroscopy method was that we were unable to make measurements in participants with heavily melanized skin. He et al., however, reported no ethnic differences in response to salt load using orthogonal polarization spectral imaging at the side of the fingers in participants of black African origin. Nevertheless, the findings reported here need to be confirmed in additional studies using techniques that are not restricted to populations with lightly melanized skin, for example orthogonal polarization spectroscopy (OPS). The numerical values of capillary densities in this cohort of healthy individuals with normal to low BP are in accordance with the published literature [8, 27–30], although meaningful comparisons with other studies are difficult due to differing techniques, BP status and smoking status. Furthermore, the data reinforce the limited information available on ranges of FCD, SCD and CR available in the literature, especially in healthy men and women older than 40 years. Changes in response to diet may differ according to sex, age and ethnic background; future studies should be designed and statistically powered to consider the influence of these inter-individual factors.

Microcirculation could be an important target to help prevent CVD or limit the progression of vascular changes, which happen very early in the development of metabolic conditions. Our findings show that adherence to DG imparted some benefit for microvascular function in a healthy population of middle-aged to older men and women, despite being a predominantly normotensive population.

## Abbreviations

CVD: Cardiovascular disease
BP: Blood pressure
DG: Dietary guidelines
CD: Capillary density
CR: Capillary recruitment
